# A Novel Metal Forming Process Based on Upsetting with Two Movable Deformation Zones Demonstrated on Railway Axle Forming

**DOI:** 10.3390/ma19122570

**Published:** 2026-06-14

**Authors:** Grzegorz Winiarski

**Affiliations:** Faculty of Mechanical Engineering, Lublin University of Technology, 38 D Nadbystrzycka Str., 20-618 Lublin, Poland; g.winiarski@pollub.pl

**Keywords:** stepped shaft, metal forming, upsetting, extrusion, railway axle

## Abstract

This paper presents a new process for forming stepped shafts by upsetting with two movable deformation zones. The developed technology enables several shaft steps to be formed at the same time, thereby increasing process efficiency and reducing material consumption. A distinctive feature of the process is that it uses two forming sleeves, each with a variable cross-section of the impression, which move in an opposite direction to that of the punches during operation. This results in a simultaneous occurrence of upsetting and extrusion, thus leading to intensified plastic deformation and stabilized metal flow. The practical applicability of the process is demonstrated on the example of a forged railway axle. An analysis is carried out by the finite element method (FEM) using specimens of hot-formed C35 steel. The obtained results reveal proper material flow and the correct filling of the tool impressions. The examination of strain and stress distributions confirms favorable forming conditions. The calculated values of the Cockcroft–Latham integral indicate favorable forming conditions and a low risk of fracture initiation during the analyzed process. The results demonstrate the potential of the proposed technology and provide a basis for future experimental verification and industrial assessment.

## 1. Introduction

The manufacturing of stepped shafts constitutes an important research area in metal forming due to the widespread application of such components in the machinery, energy, transportation, and other industrial sectors. A particular group of these components includes railway axles. These parts are an important subject of research in manufacturing technologies due to their operational significance and stringent requirements for reliability, fatigue strength, and static strength. The literature reports various technologies for forming stepped shafts, including cross wedge rolling, skew rolling, flow forming, rotary swaging, forging, rotary compression, and extrusion. These processes are aimed at producing components with high geometric accuracy, favorable microstructure, and appropriate mechanical properties. Individual technological approaches differ in terms of deformation mechanisms as well as stress and strain states, which directly affects the final geometric and mechanical properties of products.

Cross wedge rolling (CWR) is a process in which the material is formed using wedge-shaped tools that simultaneously induce rotation and elongation of the workpiece. The process enables the manufacture of axisymmetric stepped components with relatively high efficiency and favorable fiber orientation. The available literature on CWR demonstrates various approaches to the forming of stepped shafts and railway axles, including tool design modifications, process parameter analysis, and product quality assessment [1,2]. In the study in [3], the rolling process of a railway axle using a tool equipped with two wedges was analyzed; the process enabled a reduction in tool length and an increase in process efficiency. In the paper in [4], three variants of railway axle rolling were compared: conventional rolling, waste-free rolling, and rolling with a preform. Numerical analyses included material and energy consumption, force values, and susceptibility to defect formation. The risk of cracking was assessed using the Cockcroft–Latham criterion, and the numerical results were experimentally verified, confirming the possibility of producing defect-free railway axles. A subsequent study [5] investigated the possibility of manufacturing railway axles by multi-stage rolling using the same tools. The results demonstrated that high-quality products without internal and external defects could be obtained. Another study [6] focused on the influence of tool geometry, including the application of flat and convex surfaces, in the rolling process for producing railway axles. The obtained results indicated favorable distributions of temperature, strain, and damage function, with the convex tool surfaces found to be an effective technological solution. The study in [7] analyzed a new concept of railway axle rolling using long segmental tools that were moving in a similar manner to the caterpillar drive. The CWR process can also be applied to the production of shafts with gear teeth, enabling the simultaneous forming of the cylindrical section and the teeth [8]. Another important research problem is the occurrence of material fracture in CWR. For example, in the study in [9], different rolling schemes were considered, and their influence on fracture evolution was analyzed using a new hybrid fracture criterion. It was demonstrated that by changing the rolling scheme from the least favorable to the most favorable one, the risk of material fracture could be reduced by half.

Skew rolling involves deforming the workpiece by using the rolls that are inclined to the axis of this workpiece, which results in simultaneous rotation and axial motion of the material [10]. This process is applied in the manufacturing of elongated axisymmetric parts such as shafts and axles [11]. To give an example, a method for skew rolling railway axles in a three-roll mill was presented in [12]. Experimental investigations confirmed the feasibility of producing axles with high dimensional accuracy and without internal defects. Minor surface defects in the form of helical grooves were identified; however, they could be removed by machining. To eliminate the allowance required for chuck gripping, a two-stage process involving rolling extrusion and skew rolling was proposed and simulated numerically. Another study presented a comparative analysis of skew rolling in three-roll and four-roll mills for both solid and hollow railway axles [13]. Experimental results obtained for the three-roll mill showed good agreement with numerical simulations, confirming the correctness of the developed models and the applicability of this technology for railway axle production. Skew rolling processes are also subjected to numerous modifications. Research in this area focuses on solutions ensuring greater process flexibility and efficiency, such as piercing-rolling compound forming and multi-roll skew tandem rolling technology [14]. Other solutions concern the flexible skew rolling process, in which the rolls have additional degrees of freedom. The process consists of radial rolling, roll inclination, proper skew rolling, and roll withdrawal stages. Experimental investigations and numerical simulations demonstrated the possibility of manufacturing shafts with different geometries using the same tools through appropriate control of tool motion [15]. It was shown that parameters such as roll inclination angle, reduction ratio, billet wall thickness, and mandrel diameter significantly influenced the dimensional accuracy and quality of hollow products. The dimensional accuracy of hollow shafts was found to decrease with the increase in the reduction ratio, relative mandrel diameter, and roll inclination angle, while it increased with greater relative wall thickness [16].

Flow forming is a metal forming process involving the reduction in wall thickness in thin-walled tubular components by means of rollers that press the material against a rotating mandrel. This process enables high dimensional accuracy and improved mechanical properties due to strain hardening [17,18]. Research on flow forming includes investigations into new process variants. Among others, counter-roller flow-forming technology with asymmetric roller positioning was proposed [19]. It was demonstrated that roller offset affected strain distribution, process forces, and final product geometry. In other studies, the classical counter-roller flow-forming process was modified through the application of an additional container, enabling a greater reduction in billet wall thickness. This solution allowed for the generation of large strains under high hydrostatic pressure, making it possible to obtain products with an ultrafine-grained structure [20]. Another study proposed a new technology for forming internally ribbed products as an extension of the conventional rotary forming process. The use of an additional ring for restricting axial material flow ensured significantly higher rib heights [21].

Rotary swaging is a metal forming process in which the workpiece is subjected to cyclic impacts generated by the tools arranged around it. This leads to a reduced cross-sectional area and elongation of the workpiece, and the process is often performed with the simultaneous application of a mandrel to produce hollow products [22]. In the study in [23], the authors analyzed a three-stage rotary swaging process with intermediate annealing for the production of nickel aluminum bronze rods. A total area reduction of 84% was achieved, leading to significant microstructural changes and resulting in improved yield strength and elongation without deterioration of corrosion resistance. Other investigations focused on material fracture phenomena during rotary swaging. It was demonstrated that fracture features depended on deformation history and process parameters such as feed rate [24]. In another study, the rotary swaging of high-temperature alloy steel tubes for aerospace applications was analyzed with particular emphasis on the influence of annealing on the material’s microstructure and mechanical properties. It was shown that the deformed samples exhibited a greater increase in yield strength compared with tensile strength [25].

Forging is a metal forming process that is performed under cold, warm or hot conditions, enabling the production of components with favorable internal structure and high mechanical properties [26,27,28,29]. In many cases, forging processes require research into material properties in order to determine their characteristics [30,31]. In exemplary investigations [32], injection forging was proposed as an alternative to conventional multi-stage forging processes used for automotive fastener manufacturing. The results demonstrated the possibility of producing complex shapes in a single-stage process. Although higher forming forces were required, the process exhibited lower energy consumption and reduced susceptibility to material defects. Other studies proposed a two-stage cold forging process for manufacturing a drive shaft with complex geometry. The obtained products were characterized by geometry consistent with design assumptions and high dimensional accuracy [33]. Further examples of forging applications include mandrel forging and necking technology for producing hollow shafts with internal steps [34], multi-stage cold forming for manufacturing relief valve regulating nuts [35], and non-isothermal forging processes for hollow power transmission shafts [36].

Rotary compression is a process in which the workpiece is deformed by tools performing both rotational and translational motion relative to the axis of the workpiece [37]. Available research results mainly concern the production of stepped hollow components which may additionally contain gear teeth. In this case, the process is usually carried out in two stages. During the first stage, a cylindrical area reduction is made on the workpiece, while the second stage involves the formation of gear teeth on the previously reduced section. Numerous theoretical and experimental studies have confirmed the application potential of this technology [38,39].

Extrusion is a process enabling the production of solid and hollow components with constant or variable cross-sections [40]. In addition to the conventional extrusion variants, complex methods are being developed as alternatives to traditional manufacturing technologies. Examples include upsetting with a controllable deformation zone [41,42], extrusion with a movable sleeve [43], simultaneous extrusion with two movable dies [44], and incremental radial extrusion [45].

The above literature review indicates that a wide range of technologies is currently applied in the manufacturing of stepped shafts. Each of the analyzed methods enables the production of components with appropriate geometric and material properties, with process parameters and tool geometry playing a particularly important role. At the same time, these processes are being continuously developed through the introduction of new technological variants, parameter optimization, and the integration of different forming methods. In response to the increasing requirements regarding product quality, efficiency, and manufacturing flexibility, a further development of metal forming technologies is necessary, including both modifications of the existing processes and the development of new methods for stepped shaft forming. To address these needs, a new forming technology is proposed, and its assumptions and application potential are presented in this paper using the example of railway axle formation. The proposed solution is consistent with current trends in the development of advanced forming technologies. As a novel forming concept, its technological feasibility and potential applicability are preliminarily evaluated in the present study by means of finite element simulations.

## 2. Materials and Methods

**Figure 1:**
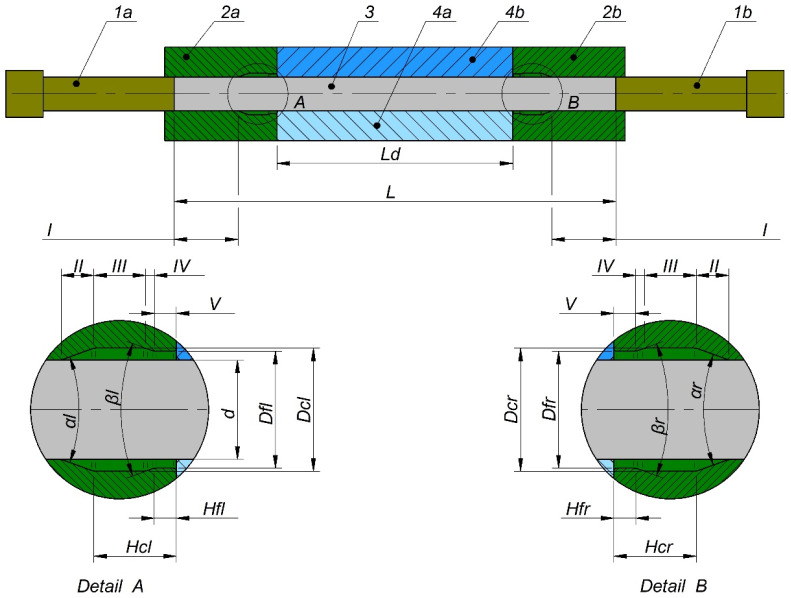
Schematic representation of upsetting with two movable deformation zones: 1a—left punch; 1b—right punch; 2a—left forming sleeve; 2b—right forming sleeve; 3—billet; 4a—lower split die; 4b—upper split die.

The new process for forming stepped shafts by upsetting with two movable deformation zones enables the efficient manufacturing of products with several area reductions (steps) having different diameters and lengths. A schematic representation of the process is shown in Figure 1. The process is carried out using a billet 3 in the form of a rod, while the die impression is formed by a tool system consisting of two punches (left 1a and right 1b), two forming sleeves (left 2a and right 2b) with variable cross-sections of their internal impression, and two split dies: lower 4a and upper 4b. At the initial stage of the process, the tools form a closed-die impression. The motion of the punches initiates material flow, resulting in the filling of the forming sleeve impressions. Once the appropriate degree of impression filling is achieved, the forming sleeves begin to move in a direction opposite to that of the punches. As a result, the initially closed-die impression opens, enabling an increase in the length of the formed shaft steps. The application of two forming sleeves makes it possible to form multi-stepped shafts without the need for intermediate operations, thereby ensuring high process efficiency through the simultaneous forming of two shaft steps.

Apart from the kinematics and relative arrangement of the tools, a distinctive feature of the process is the geometry of the forming sleeve impression. The impression of each sleeve consists of five zones. Zone I contains a hole, the diameter of which corresponds to the billet diameter. Consequently, the material does not undergo plastic deformation in this region of the tools. In the subsequent four zones, the cross-sectional area of the forming sleeve hole is significantly larger than the cross-section of the billet, which results in material deformation within these tool regions. Zone II of the sleeve impression is formed by a conical hole constituting a transition region between Zones I and III. Zone III contains a cylindrical hole with a diameter of Dcl/Dcr, which changes into a conical hole that is located in Zone IV. The final zone of the forming sleeve is a calibrating hole with a diameter of Dfl/Dfr. It should be noted that the hole diameters in Zone III are larger than those in Zones I and V. Owing to this forming sleeve geometry, the cross-section of the formed step is increased and reduced simultaneously; i.e., the upsetting of the material takes place in Zones II and III, while extrusion occurs in Zones IV and V. This intensifies the degree of plastic deformation of the material and minimizes the stress values in the regions of the workpiece located outside the die impression, i.e., between the split dies and the left and right forming sleeves, respectively. This is advantageous, as it reduces the undesirable upsetting of the material in the aforementioned regions. It should also be emphasized that depending on the zone, the forming sleeve impression can have a non-circular cross-section, which enables the production of steps with various cross-sectional shapes, such as hexagonal sections or toothed profiles.

An analysis of the available literature indicates that to date, no other authors have proposed a process for forming stepped shafts using two movable deformation zones created by forming sleeves that move in an opposite direction to that of the punches. In particular, no solutions have been identified that combine the counter-motion of the forming sleeves and punches with a simultaneous execution of upsetting and extrusion operations in two regions of the workpiece. Therefore, the technology proposed in this study represents a novel method for forming multi-step shafts and axles, one that is characterized by different material flow conditions and offers new technological capabilities compared with previous methods.

The key kinematic parameters of the process are the appropriate velocities of the punches and forming sleeves. These velocities must be properly selected, and their values can be determined based on the principle of constant volume. The volume of material leaving Zone I in the forming sleeve impression must be equal to the volume of material leaving Zone V of the tool impression. Accordingly, the velocities of the forming sleeves are strictly dependent on the punch velocities and the impression dimensions, which can be described by relationships (1) and (2) for a case when the holes in individual zones of the forming sleeve are cylindrical.

(1) $$vcr=vpr\cdot \frac{d^{2}}{{Dfr}^{2}-d^{2}},$$

(2) $$vcl=vpl\cdot \frac{d^{2}}{{Dfl}^{2}-d^{2}},$$

where:

*vcr/vcl*—velocity of the right/left forming sleeve;

*vpr/vpl*—velocity of the right/left punch;

*d*—diameter of the forming sleeve hole in Zone I;

*Dfr/Dfl*—diameter of the hole in the right/left forming sleeve in Zone V.

Other important process parameters include the values of the reduced cross-section in the forming sleeves (between Zones III and V), as well as the assumed diameter ratio in the right and left step regions. These relationships can be described by Equations (3)–(6), which apply to a case when the holes in individual zones of the forming sleeve are cylindrical.

(3) $$\delta_{r}=\frac{{Dcr}^{2}-{Dfr}^{2}}{{Dcr}^{2}}\cdot 100\%,$$

(4) $$\delta_{l}=\frac{{Dcl}^{2}-{Dfl}^{2}}{{Dcl}^{2}}\cdot 100\%,$$

(5) $$\kappa_{r}=\frac{Dfr}{d}\cdot 100\%,$$

(6) $$\kappa_{l}=\frac{Dfl}{d}\cdot 100\%,$$

where:

*δ_r_*/*δ_l_*—cross-sectional reduction in the billet in the right/left forming sleeve;

*κ_r_*/*κ_l_*—diameter ratio in the right and left step regions.

Due to its innovative character, the proposed technology requires extensive investigation in order to comprehensively evaluate its capabilities and implementation potential. In particular, future research will focus on analyzing the influence of key process parameters on process stability and product quality, as well as identifying the relationships governing process stability and repeatability. At the same time, an important research direction will involve determining potential application areas for the proposed solution. Considering the above, the present study focuses on the practical applicability of the developed technology using the example of railway axle formation, which enables a preliminary assessment of the method’s suitability for the transportation industry and indicates directions for further research and process optimization.

**Figure 2:**
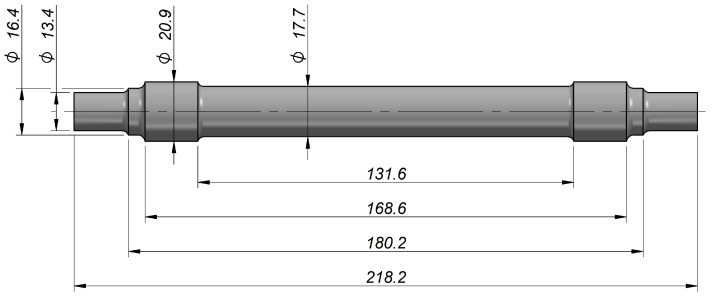
Basic geometric dimensions of a railway axle at a scale of 1:10.

Figure 2 shows the basic geometric dimensions of the analyzed railway axle which constitutes the reference model for further investigations. The analyses were carried out using a 1:10 scale model. This approach not only preserves the essential geometric and mechanical characteristics of the real component but also significantly reduces the computational time of numerical simulations and enables the application of a finite element mesh with a smaller element size. Consequently, the accuracy of the obtained results can be increased while simultaneously reducing computational resource requirements.

It was assumed that the forming of a railway axle would begin with upsetting using two movable deformation zones, according to the scheme presented in Figure 1. The key dimensions of the tool are presented in Table 1. It was assumed that the billet diameter was slightly larger than the diameter of the axle in its central region, due to machining allowance. Consequently, the diameters of the end steps of the analyzed part are smaller than the diameter of the billet. For this reason, the new upsetting process was supplemented with two open-die extrusion operations in order to obtain the final shape of the product. These operations were carried out using dies whose schematic drawings and basic dimensions are presented in Table 2. Both operations were performed assuming a cross-sectional reduction of approximately 20% and a die velocity of 100 mm/s.

The adopted process for producing a railway axle was verified by means of numerical simulations by the finite element method (FEM). The billet was modeled as a rigid-plastic object, while four-node tetrahedral elements were used for its discretization. The billet was discretized using a finite element mesh consisting of 117,555 elements. The tools were modeled as rigid objects. Remeshing was performed using the relative geometric criterion available in the software program. The procedure was triggered automatically when the ratio of the distance between the midpoint of an element edge and the die surface to the initial length of that edge exceeded the threshold value of 0.7. The finite element equations were solved using a Conjugate Gradient solver. C35 steel was selected as the deformable material, and its material model was taken from the database of the Deform 3D v11 software program used for the simulations. The material flow curve was defined in a tabular form containing flow stress data for different temperatures and strain rates. It was assumed that the process would be carried out under hot-forming conditions. Since the central part of the billet does not undergo deformation, it was assumed that only the end sections of the billet, each with a length of 60 mm, would be heated to a temperature of 1100 °C. The remaining part of the billet was assumed to have a temperature of 20 °C. The temperature of the tools was assumed to be 200 °C. The contact conditions between the deformable material and the tools were modeled using a shear friction model with a friction factor of 0.3. In this model, friction is a function of the yield stress of the deforming material. The heat transfer coefficient between these bodies was assumed to be 20 kW/m^2^K. Due to the fact that the steps on a railway axle were identical on both sides of the part, the same punch velocity of 100 mm/s was assumed for the left and right punches. In addition, the same forming sleeve velocity of 258.3 mm/s was applied for the left and the right forming sleeve. It was assumed that one simulation step corresponded to a punch displacement of 0.1 mm.

**Figure 3:**
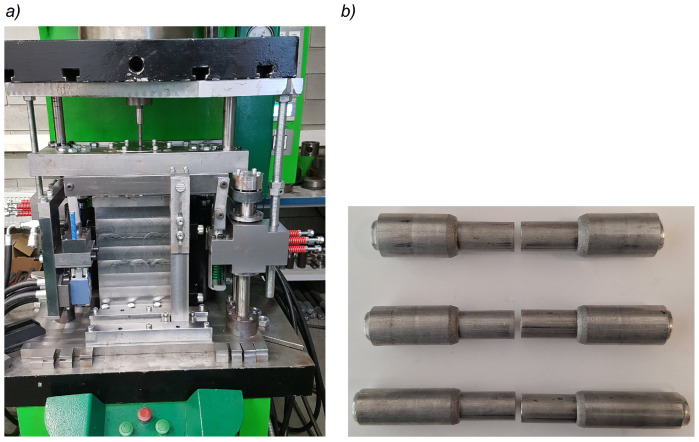
Experimental setup used for upsetting with one movable deformation zone: (**a**) experimental device; (**b**) examples of forged parts obtained in the process.

The results presented in this study were obtained from numerical analyses performed using the finite element method. It should be emphasized that the analyzed process concerns a novel upsetting technology with two movable deformation zones, for which no dedicated equipment or technological devices currently exist to enable experimental investigations. Experimental verification would require the prior design and manufacture of a specialized forming machine, which is beyond the scope of the present study. At the same time, the theoretical basis for the presented analysis comes from the author’s previous investigations on upsetting with one deformation zone of variable position, described in detail in the author’s monograph [46]. For that process, a dedicated experimental device was designed and manufactured, and the obtained results were verified both numerically and experimentally. Photographs of the dedicated experimental device installed in the working space of a universal hydraulic press, as well as selected forgings produced using the upsetting process with one movable deformation zone, are presented in Figure 3. Good agreement between the numerical and experimental results was obtained, confirming the correctness of the adopted numerical models, boundary conditions, and calculation methodology. The process analyzed in the present study is a development of the upsetting technology with one deformation zone of variable position; it is based on analogous metal flow phenomena while maintaining similar kinematic and energy conditions of the process, as well as comparable nature of strain and stress distributions in the deformed material. Therefore, it can be assumed that the previously validated assumptions would remain reliable also in the case of upsetting with two movable deformation zones. Consequently, the presented numerical results may be considered a reasonable representation of the expected process behavior and may provide a basis for further design work and experimental investigations. Therefore, the numerical results presented in this study should be regarded as a preliminary assessment of the technological feasibility of the proposed process rather than a full validation of its industrial applicability.

## 3. Results and Discussion

**Figure 4:**
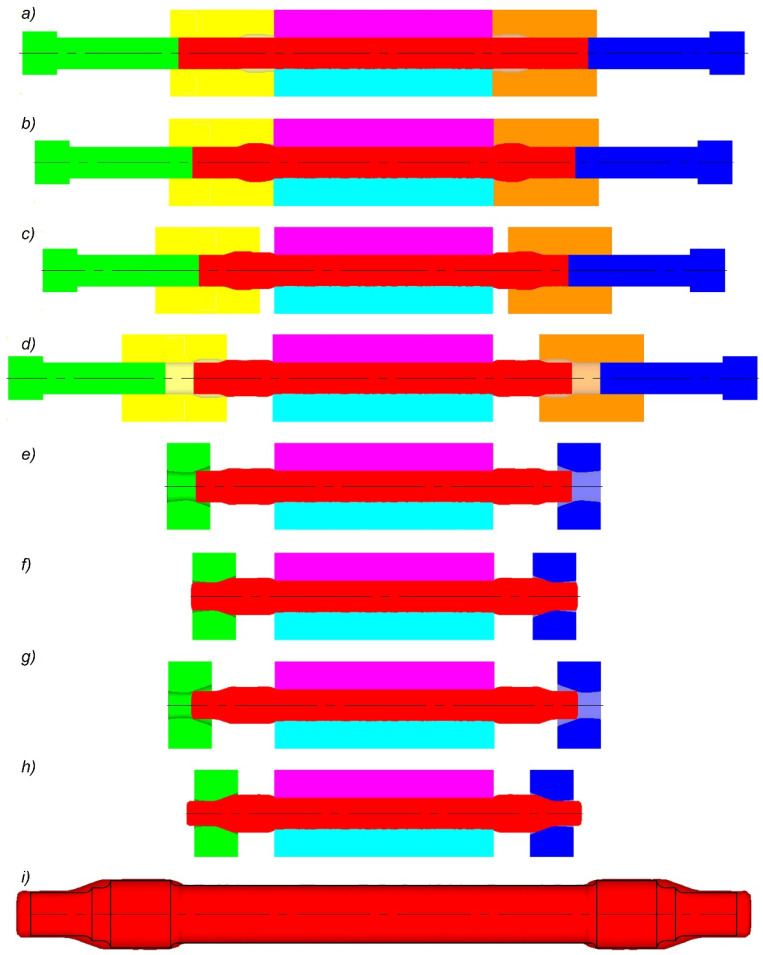
Shape progression of a railway axle; description provided in the text (**a**–**i**).

Figure 4 shows changes in the shape of a railway axle. The process begins with the motion of the punches, causing the billet material to fill in the impressions of the left and right forming sleeves (Figure 4a,b). Subsequently, the forming sleeves begin to move in a direction opposite to that of the punches, which enables an increase in the length of individual steps on the workpiece (Figure 4c). After the required step lengths are achieved, the punches and forming sleeves are withdrawn, while the deformation of the workpiece continues (Figure 4d). This stage constitutes the final phase of upsetting with two movable deformation zones. In subsequent operations, the steps on railway axle ends are formed. This is carried out via a two-stage open-die extrusion process. During these operations, only the dies move, causing a gradual reduction in the cross-section in specified regions (Figure 4e–h). The final shape of the part together with the outline of the finished product are presented in Figure 4i. The diameter of the largest steps ranges from 21.6 mm to 21.9 mm, while the diameter of the steps on railway axle ends is 14.4 mm. The obtained forging has allowances for finishing machining, which is in accordance with the design requirements. The numerical results indicate that it should be possible to obtain a railway axle with the assumed geometry and dimensions.

**Figure 5:**
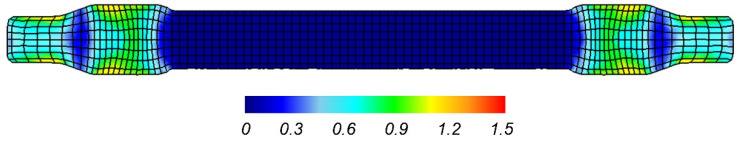
Distribution of effective strain in the axial cross-section of a railway axle, together with the coordinate grid pattern.

A view of the coordinate grid in the axial section of the workpiece illustrating the material flow lines together with the distribution of effective strain is presented in Figure 5. The grid lines exhibit a correct pattern and are arranged parallel to the external contour of the product. In the regions of the steps with the largest diameter, one can observe a densification of the grid lines in the axial direction and an increase in the spacing between these lines in the radial direction. On the other hand, in the regions of the end steps with the smallest diameter, the spacing between the grid lines increases in the axial direction and decreases in the radial direction. This is consistent with the adopted technology for forming individual steps on the workpiece. The concentration of effective strain occurs in the regions of the steps with the largest and the smallest diameters. In the case of the largest-diameter steps, the distribution of the analyzed parameter is similar to that observed during the upsetting of a cylindrical specimen between flat dies. It can be observed that the effective strain values are lower near the end regions of these steps, while higher values occur in the central region of the workpiece. The maximum values of approximately 1.5 are located near the external surface of the product. As for the end steps of the forging, the effective strain values are distributed in a layered manner. The lowest values occur near the axis of symmetry and increase toward the external surface of the part. In the central step of the forging, the material does not undergo plastic deformation because it is maintained within the closed impression formed by the split dies.

**Figure 6:**
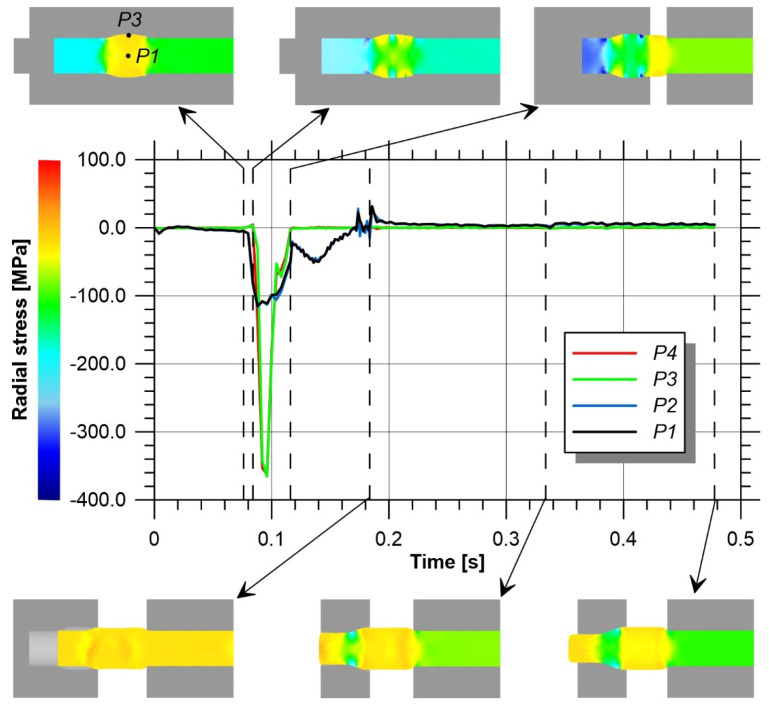
Variation in radial stress values at the analyzed points on a railway axle forging.

Next, the stress state was examined at four selected points on the workpiece. Prior to the process, two points on the left side of the billet (i.e., points P1 and P3) were defined at a distance of 10 mm from the front surfaces of the lower and upper split dies, respectively, toward the left punch. The first point, P1, was located on the billet symmetry axis, whereas the third point, P3, was on its lateral surface (Figure 6). Points P2 and P4 were located on the right side of the billet, in the same manner as P1 and P3. During the process, all points moved together with the deformed material. The locations of measuring points P1–P4 were selected to represent both the axial and surface regions of the material in the areas that are particularly important from a viewpoint of dimensional accuracy and process stability. The points were positioned near the split die, where the material remains outside the tools for most of the process and is not therefore directly constrained by the tool impression. As a consequence, these regions are potentially the most susceptible to uncontrolled material flow and dimensional deviations from the target geometry. By monitoring the stress state at these locations, it is possible to assess the likelihood of undesirable material upsetting outside of the tool impression and to verify the effectiveness of the proposed forming method in terms of the required dimensions of the part. In addition, the symmetric arrangement of the points on both sides of the workpiece enables the verification of the symmetry of material flow and stress distribution during the process.

Figure 6 presents the variation in the radial stress values at the analyzed measuring points on a railway axle forging. Up to 0.076 s of the process duration, the impressions of the left and right forming sleeves are being filled; therefore, the deformed material does not yet come into contact with Zone III of the sleeve hole. For this reason, the radial stress values at the measuring points oscillate around zero. As the upsetting with two movable deformation zones proceeds, the material’s radial flow becomes constrained by the forming sleeve hole in Zone III. In effect, compressive radial stresses occur. After the forming sleeves are set in motion, the maximum values of the analyzed parameter are observed. On the lateral surface of the workpiece, the radial stress reaches approximately −360 MPa, whereas on the symmetry axis of the product it amounts to about −120 MPa. The final stage of the upsetting with two movable deformation zones takes place from 0.116 s to 0.183 s, during which the punches and forming sleeves are withdrawn. The analyzed points are no longer located within the tool impression and thus no longer constrained by the tool surfaces. Consequently, the radial stress decreases again to nearly zero. During the first and second open-die extrusion operations, i.e., from 0.183 s and 0.333 s, the analyzed measuring points are still beyond the tool; therefore, the radial stress remains close to zero. It can also be observed that the stress variation curves for the corresponding points on the left and right sides of the part are nearly identical, which results from the symmetry of the analyzed part.

**Figure 7:**
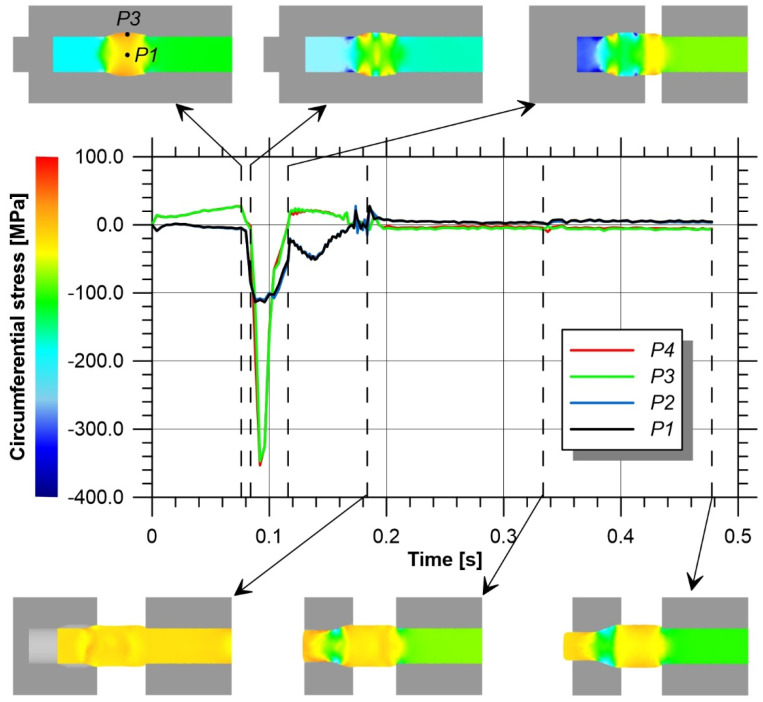
Variation in circumferential stress values at the analyzed points on a railway axle forging.

Figure 7 presents the variation in the circumferential stress values at the analyzed measuring points on a railway axle forging. Until the deformed material comes into contact with Zone III of the forming sleeve hole (0.076 s), the circumferential stresses in the axis of the workpiece oscillate around zero, whereas tensile circumferential stresses reaching up to 40 MPa occur on its outer surface. It can be observed that at this stage of the process, the stress values increase as the process progresses due to an increase in the diameter of the formed steps. The contact of the material with the aforementioned Zone III of the forming sleeve changes the stress state and leads to the occurrence of compressive circumferential stresses with their maximum values of approximately −120 MPa and −360 MPa in the axis of the workpiece and on its outer surface, respectively. In the final stage of upsetting with two movable deformation zones (i.e., from 0.116 s to 0.183 s), a non-uniform stress state is observed at the analyzed measuring points. In the axis of the workpiece, the circumferential stresses are compressive, whereas tensile stresses occur on the outer surface of the workpiece. During the open-die extrusion operations, the circumferential stresses at the analyzed measuring points oscillate around zero. This results, among others, from the fact that the cross-section of the step formed during these operations is significantly smaller than the cross-section of the step where the measuring points are located. Therefore, higher stress values occur in the steps with smaller diameters, which is consistent with the adopted forming technology for railway axles.

**Figure 8:**
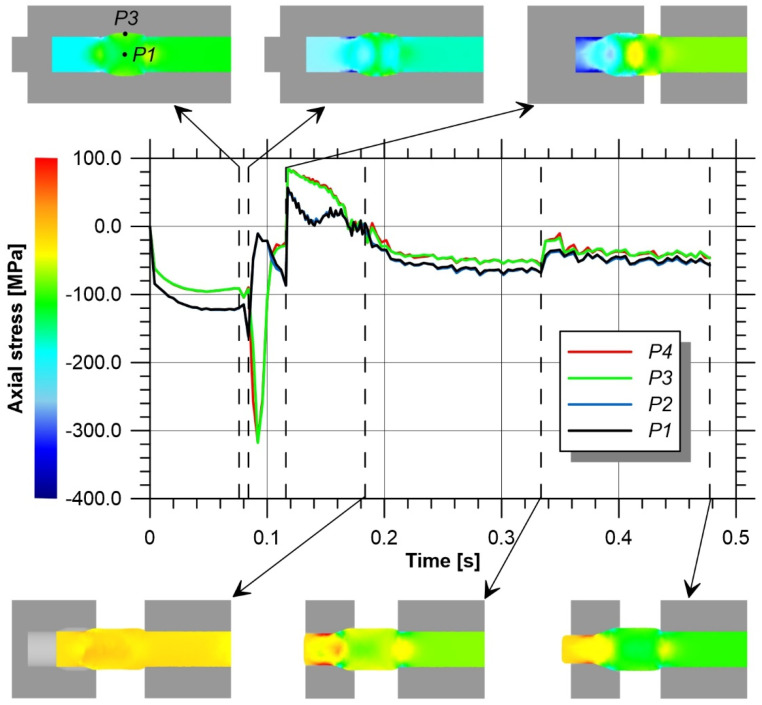
Variations in axial stress values at the analyzed points on a railway axle forging.

Figure 8 presents the variation in the axial stress values at the analyzed points on a railway axle forging. It can be observed that until the end of the main stage of upsetting with two movable deformation zones, during which the punches and forming sleeves move in opposite directions (i.e., up to 0.116 s), the axial stresses are compressive in nature. During this stage, the motion of the punches causes axial compression of the material and a reduction in billet length, which generates the axial compressive stresses. During the final stage of the process, when the forming sleeves and punches are withdrawn (from 0.116 s to 0.183 s), axial tensile stresses are induced in the material. These stresses are higher at the points located on the outer surface of the workpiece. In contrast, axial compressive stresses occur at all measuring points during both open-die extrusion operations. The values of these stresses are sufficiently low, so they do not cause a significant increase in the diameters of the steps in these regions of the part. Such stress distribution during the open-die extrusion operations is required to avoid the undesirable upsetting of the material in front of the die.

**Figure 9:**
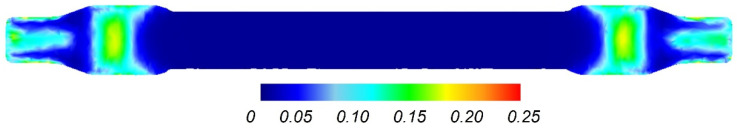
Distribution of the normalized Cockcroft–Latham fracture criterion values in the axial cross-section of a railway axle forging.

The risk of material cohesion loss was assessed based on the normalized Cockcroft–Latham fracture criterion. The distribution of this parameter in the axial cross-section of the part is presented in Figure 9. The maximum values of the integral reach 0.25 and are considered safe for the analyzed material grade under hot working conditions. The highest values are located in the regions of the steps with the largest diameters, near the axis of symmetry. In the end steps of the part, the maximum values of the integral occur both in the axis of the axle and near its outer surface. The obtained values of the Cockcroft–Latham integral indicate favorable forming conditions and a low risk of fracture initiation in the numerical model. However, it should be emphasized that a definitive assessment of the material’s susceptibility to cracking would require experimental validation of the simulated process and calibration of the applied fracture criterion.

**Figure 10:**
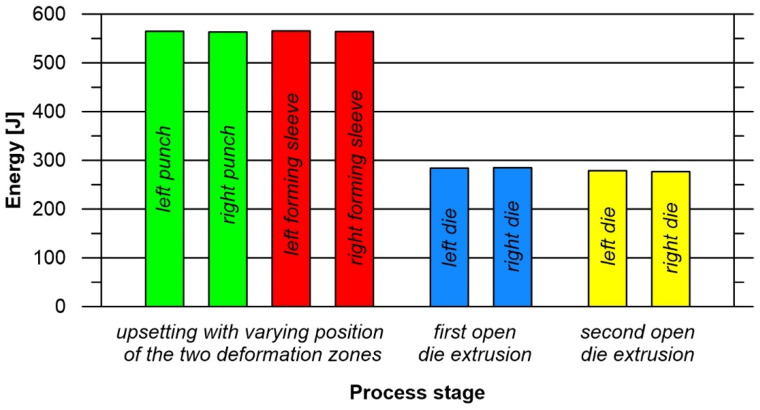
Energy consumption in the analyzed process for forming a railway axle.

Figure 10 presents the results of energy consumption during the forming process for a railway axle. The upsetting process with two movable deformation zones is characterized by a nearly identical energy demand for individual tools. Both the left and right punches, as well as the left and right forming sleeves, perform similar work at a level of approximately 560 J each. In the case of both open-die extrusion operations, individual dies perform a work of approximately 280 J. Consequently, the formation of the largest diameter steps by the proposed method requires a total tool work of approximately 2240 J, whereas for the work required for forming the steps on the workpiece ends must be approximately 1120 J.

## 4. Conclusions

The numerical analyses confirmed the feasibility of applying the new upsetting process with two movable deformation zones for the forming of multi-stepped axles and shafts. The numerical results indicate that the developed technology may enable simultaneous formation of several steps of the workpiece while maintaining favorable material flow conditions and high process stability. It was demonstrated that the application of two forming sleeves with appropriately designed geometries allowed for the simultaneous realization of upsetting and extrusion operations, which led to intensified plastic deformation and limited the undesired upsetting of the material outside of the tool impression. The opposite movement of the forming sleeves relative to the punches enabled controlled elongation of the formed steps and ensured the stability of the process. The obtained results indicate that the developed technology holds considerable potential for industrial application in the manufacturing of stepped shafts with complex geometries. The proposed solution may provide a basis for further experimental investigations, process parameter optimization, and the development of dedicated technological equipment enabling industrial implementation of the new forming method.

The analysis performed for a railway axle forging demonstrated that the proposed technology might constitute an alternative to conventional forming methods for axisymmetric products, particularly in terms of components characterized by complex geometries and significant differences in step diameters. Based on the results, the following conclusions were formulated:

- The proposed forming method ensures correct material flow, the proper filling of the tool impression, and the production of a railway axle with the assumed geometry and dimensions.
- The highest effective strain values were located in the regions of the steps with the largest and the smallest diameters.
- The stress analysis revealed the predominance of compressive stresses during the key stages of the process, which favorably reduced the risk of material fracture.
- The calculated Cockcroft–Latham integral values indicate the low risk of material fracture initiation under the analyzed forming conditions.
- The process is characterized by uniform energy demand for the moving tools during the main forming operation.
- The developed technology appears promising for future industrial applications; however, experimental validation is required before its practical implementation can be fully assessed.
- Since the present study is based exclusively on finite element simulations, the obtained results should be treated as a preliminary numerical verification of the proposed concept.

## Figures and Tables

**Table 1:** Dimensions of a tool used in upsetting with two movable deformation zones (designations according to Figure 1).

Ld [mm]	L [mm]	D [mm]	Dfr [mm]	Dfl [mm]	Dcr [mm]	Dcl [mm]
126	236	18	21.2	21.2	22.35	22.35
Hcr [mm]	Hcl [mm]	Hfr [mm]	Hfl [mm]	δ_r_ [%]	δ_l_ [%]	
15	15	4	4	10	10	
*κ*_r_ [%]	*κ*_l_ [%]	αr [°]	αl [°]	βr [°]	βl [°]	
117.7	117.7	40	40	40	40	

**Table 2:** Dimensions of a tool used in open-die extrusion processes.

	Operation I	Operation II	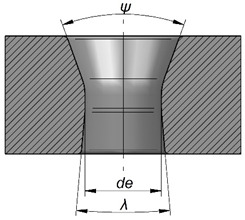
de [mm]	16.2	14.5	
ψ [°]	40	40	
λ [°]	10	10	

## Data Availability

The original contributions presented in this study are included in the article. Further inquiries can be directed to the corresponding author.
